# Recent Advances in Antifreeze Peptide Preparation: A Review

**DOI:** 10.3390/molecules29204913

**Published:** 2024-10-17

**Authors:** Bo Xia, Juntao Wang, Honghao Chen, Shuyan Lin, Buchun Pan, Nan Wang

**Affiliations:** Department of Bioenvironment, Jiyang College of Zhejiang A&F University, Zhuji 311800, China

**Keywords:** antifreeze agents, antifreeze peptide, sub-zero storage

## Abstract

Antifreeze agents play a critical role in various fields including tissue engineering, gene therapy, therapeutic protein production, and transplantation. Commonly used antifreeze agents such as DMSO and other organic substances are known to have cytotoxic effects. Antifreeze proteins sourced from cold-adapted organisms offer a promising solution by inhibiting ice crystal formation; however, their effectiveness is hindered by a dynamic ice-shaping (DIS) effect and thermal hysteresis (TH) properties. In response to these limitations, antifreeze peptides (AFPs) have been developed as alternatives to antifreeze proteins, providing similar antifreeze properties without the associated drawbacks. This review explores the methods for acquiring AFPs, with a particular emphasis on chemical synthesis. It aims to offer valuable insights and practical implications to drive the realm of sub-zero storage.

## 1. Introduction

Water is a fundamental element within the cellular composition of all living organisms on Earth, playing a crucial role in sustaining essential life processes. While water’s significance is undeniable, cells face the risk of damage from ice crystal formation, especially at low temperatures, which can ultimately result in cell death [1]. To overcome this risk, antifreeze agents like DMSO and other organic substances are commonly used, albeit with the drawback of potentially causing cytotoxic effects [2]. Thankfully, nature has devised another solution in the form of antifreeze proteins found in the cells of organisms inhabiting cold regions. These proteins can interact with the surface of ice crystals, effectively inhibiting their formation and growth [3]. The revelation of antifreeze proteins has piqued the curiosity of researchers across a spectrum of fields, from organ transplantation to cell cryopreservation and frozen food preservation [4,5,6]. However, with time, antifreeze proteins have exhibited certain limitations. Primarily, their high cost presents significant challenges [7]. Furthermore, numerous studies in the realm of low-temperature storage have indicated that the mere addition of antifreeze proteins may not substantially enhance cell recovery rates. This limitation has been attributed to the dynamic ice-shaping (DIS) effect associated with antifreeze proteins, stemming from their thermal hysteresis (TH) properties [8,9]. Under the influence of DIS, antifreeze proteins tend to bind selectively to specific ice crystal surfaces, triggering varying growth rates among different crystal facets and resulting in diverse ice crystal morphologies. Critically, the growth of ice crystals on inhibited crystal planes may induce mechanical stress, leading to needle-like crystal structures that could harm cells [10].

**Scheme 1:**
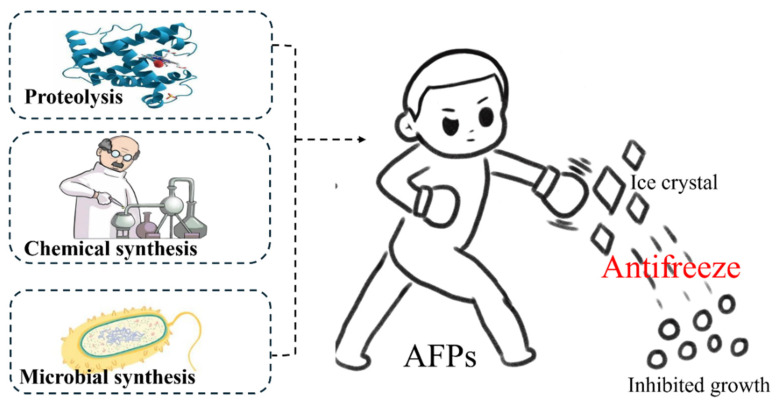
Main route for preparing AFPs.

To address these challenges, scientists have developed derivatives of antifreeze proteins known as antifreeze peptides (AFPs). AFPs offer similar antifreeze properties without the TH and DIS effects, binding strongly to all ice crystal faces and mitigating the negative impact of DIS [11,12,13]. AFPs have shown great promise as biological antifreeze materials, offering excellent antifreeze properties, biocompatibility, and physiological activities. To date, there are three main pathways for obtaining AFPs: proteolysis of antifreeze proteins from ocean fish in cold regions, chemical synthesis, and microbial synthesis (Scheme 1). Among these methods, proteolysis currently predominates. However, it is still encountering certain obstacles, such as random hydrolysis sites, an unmodifiable peptide sequence, and a high reliance on marine organisms. The presence of random hydrolysis sites and an unalterable peptide sequence often result in a mixture of peptides that are unsuitable for specific applications. Furthermore, the heavy dependence on marine organisms can exacerbate environmental pressures in the ocean. Therefore, chemical and microbial synthesis are deemed the most viable solution for future AFP production. This review explores the methods for acquiring AFPs, with a particular emphasis on chemical synthesis. It aims to offer valuable insights and practical implications to drive the realm of sub-zero storage.

## 2. Formation of Ice Crystals and Their Damage to Cells

Cryopreservation has become an essential technique in a variety of cell-based applications, including stem cell therapy, tissue engineering, human-assisted reproduction, and transfusion medicine [14,15,16,17]. By cooling living materials such as food, biological organs, and tissues to low temperatures, cryopreservation reduces spoilage caused by bacterial growth, plant respiration, and microbial activity. However, when aqueous solutions are cooled below the equilibrium freezing point, ice crystals inevitably form in the extracellular medium. This process reduces the water concentration within the sample, leading to potential cell damage. The formation of ice crystals, particularly in the extracellular space, can cause severe mechanical stress on cell membranes and result in cellular damage or necrosis [18]. This section will explore the underlying mechanisms of ice crystal formation and how it contributes to cell damage in subfreezing environments.

### 2.1. Formation of Ice Crystals

During cryopreservation, ice crystals form spontaneously in water-containing biological materials once temperatures drop below freezing due to supercooling. Ice formation consists of two key phases: crystallization and recrystallization, both of which are influenced by the freezing rate and storage temperature. The freezing rate determines the size and distribution of ice crystals, while storage temperature controls the total number of crystals [19]. The characteristics of ice crystals, such as size, morphology, and spatial distribution, are dictated by the interplay between nucleation and crystal growth [20]. Theories explaining intracellular ice formation include the pore theory, which suggests that extracellular ice seeds intracellular ice formation through pores in cell membranes. Other explanations involve surface-catalyzed nucleation, where the cell membrane promotes ice formation, or volume-catalyzed nucleation, where intracellular particles act as catalysts [21]. Another theory is the cell membrane damage theory, which posits that extracellular ice seeds intracellular ice via membrane damage caused by electrical transients or osmotic pressure.

#### 2.1.1. Ice Crystallization

Supercooling occurs when the temperature is lowered below the freezing point without inducing solidification, and supersaturation refers to a state where solute concentration exceeds equilibrium solubility. Together, these conditions allow crystallization, which starts with the formation of ice crystals and is followed by their growth. D. Zaragotas et al. [22] developed a platform using infrared video recordings to monitor ice nucleation events in a 96-well microplate. Their temperature data analysis identified freezing curves that were classified as either fast or slow, with nucleation, freezing, and solid-phase points marking the crystallization process’s key phases.

(a) Nucleation of ice crystals

**Figure 1:**
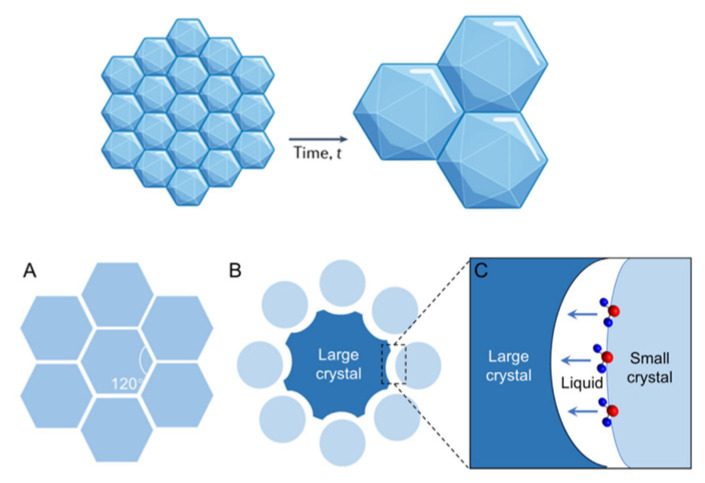
Ice crystallization process: (**A**) The intersection angles at the triple junctions are 120° if the ice crystals are the same size. (**B**) Large crystals tend to have concave boundaries and small crystals tend to be convex. (**C**) Schematic of boundary migration of adjacent ice crystals.

At sub-zero temperatures, two competing phenomena—supercooling and ice nucleation—drive the cellular response. Supercooling refers to a metastable state where water remains liquid below its freezing point, with the help of antifreeze proteins that enhance supercooling and result in smaller ice crystals [20,22]. Once nucleation begins, it interrupts the supercooled state, initiating freezing [22]. Nucleation is influenced by factors like melting temperature, ice–liquid interfacial tension, and kinetic factors. In supercooled liquids, molecular vibrations lead to the spontaneous formation of ordered clusters of solid molecules, or ice nuclei [21]. Nucleation is crucial for freezing rate instability and crystallization. During this phase, a supercooled liquid transitions to a two-phase liquid–solid state, releasing latent heat of freezing (Lf) (Figure 1B). The temperature rise from the nucleation point (np) to the freezing point (fp) defines the degree of supercooling (∆Tsc), marking the dendritic phase [22]. Classical Nucleation Theory (CNT) is often used to explain this process [21,23].

(b) Growth of ice crystals

Once nucleation occurs, ice crystals grow as free energy decreases. This growth starts at the freezing point (fp) and continues until the solid phase line point (sp), marking the final stage of crystallization. Ice crystal growth can be described using a non-isothermal, diffusion-limited growth model [21,22]. The solid phase line represents the completion of crystallization as the sample transitions to a single-phase solid state. At this point, latent heat release stops, and the temperature decreases linearly until reaching equilibrium with the cooling medium [22].

#### 2.1.2. Ice Recrystallization

Ice recrystallization, a form of Ostwald ripening, occurs when larger ice crystals grow at the expense of smaller ones to reduce surface energy. This process increases ice crystal size and contributes to cell death during thawing, adding osmotic stress [18,24]. In reality, ice crystals differ in size and curvature; larger crystals have concave boundaries with lower surface energy, while smaller crystals have convex boundaries (Figure 1). According to Fick’s first law of diffusion, atoms migrate from areas of high curvature to low curvature, causing smaller crystals to shrink and larger ones to grow [21]. As storage temperatures rise slightly, smaller crystals melt more rapidly, and their meltwater reforms around larger ice crystals, leading to mechanical cell damage and osmotic stress [18].

### 2.2. Ice Crystal-Induced Cell Damage

**Figure 2:**
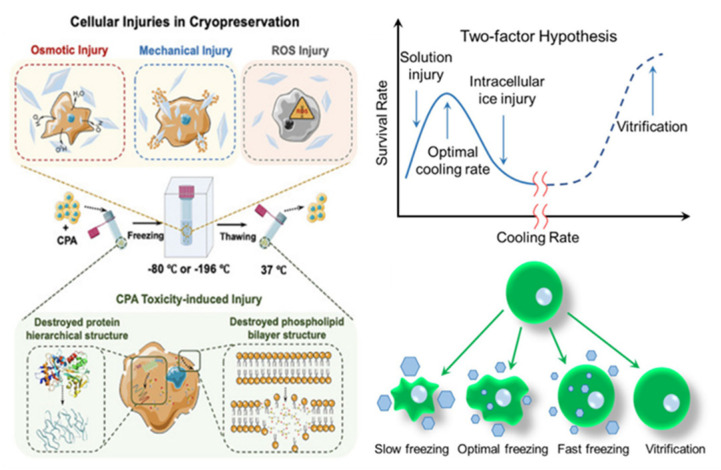
Cellular injuries in cryopreservation.

At ultra-low temperatures (below −130 °C), the kinetic energy and molecular motion within biological materials significantly decrease. This reduction slows chemical and biological reactions, including metabolism, active transport, enzymatic activity, and diffusion, keeping the materials in a suspended state until the temperature rises again [18]. Many studies support the idea that freezing damage in slowly frozen cell suspensions is not directly caused by ice crystals but rather results from high solute concentrations that form as water is removed to create ice. This suggests that freezing damage may be due to the temperature drop itself, changes in solution composition caused by freezing, or both [21]. During the cryopreservation of biological materials, various forms of cell damage can occur, such as osmotic stress, mechanical damage, and toxicity induced by cryoprotectants (CPA) [1,25] (Figure 2).

#### 2.2.1. Osmotic Damage and CPA Toxicity-Induced Damage

The formation of extracellular ice increases osmotic pressure during freezing and thawing, leading to cellular dehydration-a process known as osmotic damage [26]. Slow cooling rates cause intracellular dehydration due to the chemical potential difference between extracellular ice and intracellular solution, a phenomenon called the solute effect [21,27]. Rapid cooling rates, however, lead to lethal intracellular ice formation due to insufficient time for water to escape, though partial dehydration may enhance cell survival. Higher cooling rates can lead to vitrification, improving the survival of frozen cells. Recrystallization during thawing can also lead to osmotic shock as solutes become concentrated between ice crystals [18]. Exposure to toxic cryoprotectants can further increase osmotic pressure, compounding damage [28].

#### 2.2.2. Mechanical Injury

During the thawing process, especially when the warming rate is slow, ice crystals can recrystallize into larger and sharper structures, which can disrupt cell membranes and lead to mechanical damage [29,30]. Intracellular ice formation (IIF) exerts mechanical stress directly on organelles and cell membranes [31]. The extent of this mechanical damage is influenced by the type and size of the ice crystals. The size of these crystals is primarily determined by the nucleation temperature—the temperature at which the first ice crystal forms [20]. Cooling rates are closely linked to the size, shape, and distribution of ice crystals: higher cooling rates increase supercooling, which reduces the grain size of intracellular ice crystals [16,27]. Ice crystals can exist in different forms (I–VI), with ice I, which forms under atmospheric pressure, having a lower density than liquid water. This leads to an expansion in crystal volume, causing damage to tissues. Under high pressure, several types of ice crystals (II–VI) form, which have a higher density than water, resulting in less damage during freezing under high hydrostatic pressure [20].

## 3. Proteolysis from Antifreezing Protein

Hydrolysis stands as one of the earliest historical techniques utilized in extracting AFPs from proteins of biological origin. This process involves enzymatic or chemical cleavage to break amide bonds within the protein structure. Through hydrolysis, a wide range of AFPs have been obtained and can be categorized into three main groups: fish-derived, insect-derived, and livestock-derived. AFPs were first discovered in the proteins of polar fish [8], showcasing sequences with a high concentration of hydrophilic groups. These specialized derivatives, known as fish-derived antifreeze peptides, have since captured considerable attention in the field. For example, the skin of Takifugu obscurus, renowned for its abundance of glycine, proline, and alanine (Ala), can be hydrolyzed using papain to yield a mixture of antifreeze peptides (TsAFP) containing three sequences (EGPRAGGAPG, GDAGPSGPAGPTG, and GEAGPAGPAG). These peptides effectively halt the growth of ice crystals and provide robust protection to muscle tissue during the freezing and thawing processes of fish mince at a concentration of 4% [32]. Silver carp is also rich in Ala, and through the use of Protamex, a novel peptide with the sequence KAADSFNHKAFFAKVG has been isolated. This peptide displays unique properties, including an α-helical structure and amphipathic characteristics, enabling it to interact with 48 water molecules simultaneously. Remarkably, this peptide has demonstrated exceptional efficacy in cryopreservation applications, achieving an impressive 90% survival rate for yeast during freezing protection [33]. The scales of Ctenopharyngodon idella, abundant in proline, exhibit a remarkable adaptability to cold-water environments. Three main antifreeze peptides (VGPAGPSGPSGPQ, RGSPGERGESGPAGPSG, and VGPAGPSGPSGPQG) with a thermal hysteresis activity of 5.09 °C have been isolated from them. Importantly, these peptides have shown a significant 84.4% recovery rate in yeast cryopreservation at −20 °C [34].

Insects, renowned for their vast species diversity, serve as abundant sources of proteins and protein-based products. The silkworm stands as a quintessential example, producing silk rich in fibrous protein widely utilized in the textile industry. In recent years, researchers have discovered that silk peptides (SKAFPs) can be obtained through enzymolysis and alkali hydrolysis, containing a plethora of hydrophilic amino acids [35]. In freezing protection experiments, the recovery rate of 1.0 mg/mL SKAFPs for *Lactobacillus delbrueckii Subsp. Bulgaricus* was 84% [36], while the recovery rate of 10 mg/mL SKAFPs for human bone-derived mesenchymal stem cells was 81% [37]. The impressive cell recovery rates achieved with insect-derived AFPs underscore the value of further exploration into natural AFPs.

**Figure 3:**
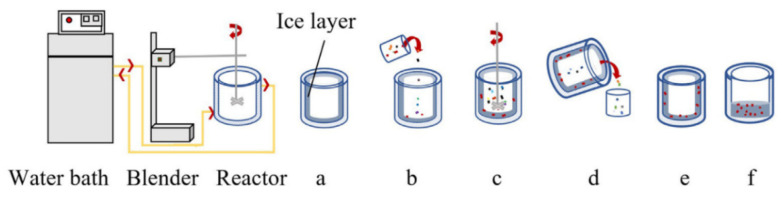
Ice affinity adsorption system.

Proteins play a vital role in the bodies of livestock, boasting diverse sequence structures. The skin and bone marrow of animals act as primary defenses against cold climates; rich in collagen protein, they are excellent candidates for the study of AFPs. For example, utilizing ice as a tool to selectively identify these AFPs, researchers extract them from bovine bone collagen protein through alkaline protease hydrolysis. This process operates akin to a magnet attracting metal with precision. These AFPs have exhibited the ability to selectively bind to various ice surfaces, resulting in altered ice crystal growth and providing protection for yeast entities within frozen dough formulations (Figure 3) [38]. In addition to cattle, pigs serve as notable sources of AFPs, preserving the quality attributes of pork loin. Intriguingly, the incorporation of 4% AFPs has been found to reduce thawing loss in frozen meat by 5.32% and decrease the area of ice crystal formation by 15.54% [39].

## 4. Chemical Synthesis of Antifreezing Peptides

While proteolysis remains prevalent, it faces challenges such as random hydrolysis sites, unmodifiable peptide sequences, and a significant reliance on marine organisms. Random hydrolysis sites and fixed peptide sequences often yield peptide mixtures unsuitable for specific applications. Additionally, the heavy dependence on marine organisms can intensify environmental pressures in the ocean. Consequently, chemical synthesis is considered the most promising solution for future AFP production due to its ability to accurately control the sequence of obtained peptides. The antifreeze properties of AFPs primarily derive from the amino acids in the peptide sequence and the peptide’s secondary structure. Therefore, in the forthcoming discussion, we will delve into the prevalent amino acids in synthesized peptide sequences, such as Ala, Pro, Thr, and other hydrophilic amino acids (refer to Table 1).

### 4.1. Antifreezing Peptides Based Ala-Dominated Sequence

**Figure 4:**
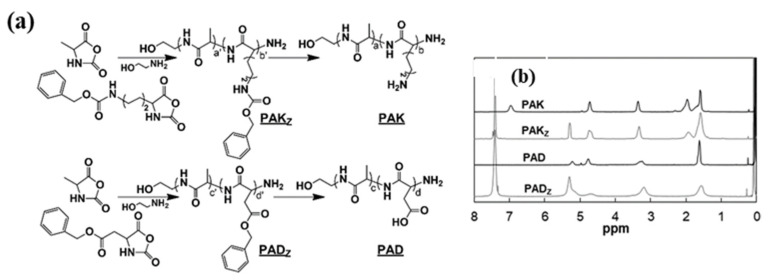
(**a**) Synthetic scheme and (**b**) ^1^H NMR spectra (CF3COOD) of the PAK and PAD (in CF3COOD).

Ala, a prominent residue found in Type I AFPs of polar fish, plays a crucial role in imparting antifreeze properties to these peptides because of its unique structural characteristics. Previous research has demonstrated the antifreeze activity of individual Ala monomers, leading scientists to explore polyalanine (PAla) [43]. The self-assembly of homologous alanine oligopeptides (DP = 3, 5, 7) represents a novel approach to producing PAla, revealing a higher specific ice-binding surface area compared to Ala and achieving approximately 17% of the Maximum Growth Limiting Size (MGLS) observed in a control group treated with PBS [44]. Beyond its spherical structure, the α-helix conformation can further enhance Ala’s antifreeze activity. Using the *N*-carboxyanhydride (NCA) polymerization method, researchers meticulously designed two distinct peptide sequences: *L*-Ala-co-*L*-lys (PAK) with ratios ranging from 0/100 to 70/30, and a random coil of *L*-Ala-co-aspartic acid (PAD) with a 1:1.1 ratio (Figure 4). Astonishingly, with increased Ala content, the structure of PAK transitioned from a random coil to a classical α-helix. This transition was accompanied by a reduction in Ice Recrystallization Inhibition (IRI) activity from 74% of PBS to 11%, with the ice-crystal coagulation temperature recorded between −22 °C and −23 °C [45,46]. However, PAD displayed no IRI activity. The introduction of hydrophilic amino acids, which can stabilize the helical structure, has been shown to enhance the antifreeze performance of AFPs predominantly composed of Ala. For instance, AFPs-p2 (DTASDAKAAAEL) incorporated hydrophilic lysine and glutamic acid into the ice-binding sites 7 and 11 of AFPs-p1 (DTASDAAAAAAL), while AFPs-p3 (DTASDAFAAAAL) introduced hydrophobic phenylalanine at site 11. The results indicated that AFPs-p3 exhibited freezing at −10.8 °C, AFPs-p1 at −11.9 °C, and AFPs-p2 achieved an impressive freezing point of −16.3 °C [47].

Ala is not solely confined to its antifreeze activity through the formation of α-helical structures. The inclusion of alternating arrangements of *L*- and *D*-configured amino acids has been shown to induce spontaneous antiparallel beta-folding in peptide chains. By employing the solid-phase synthesis (SPSS) method, researchers developed self-assembled cyclic peptides (*L*-Lys-*D*-Ala-*L*-Thr-*D*-Ala-*L*-Thr-*D*-Ala-*L*-Lys-*D*-Ala), challenging the notion that the antifreeze activity of Ala peptides is exclusively linked to α-helix formation. However, during the evaluation of the cyclic peptide’s Ice Recrystallization Inhibition (IRI) activity, a substantial amount of precipitation was encountered under basic conditions (pH = 11), leading to significantly lower IRI activity compared to that observed under acidic conditions (pH = 3), which was 24.3% of the sucrose solution MGLS [40].

### 4.2. Antifreezing Peptides Based on Proline

**Figure 5:**
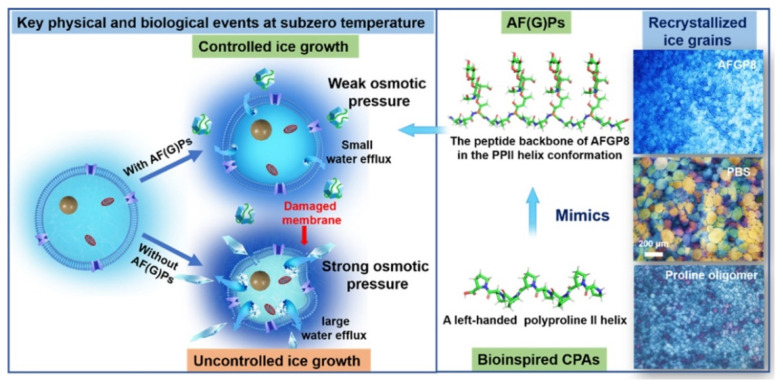
PPro is similar to the antifreeze mechanism of glycopeptide at below zero.

Glycopeptides in polar animals play a vital role in adapting to cold environments. Proline and its derivatives, the smallest mimetics of antifreeze glycopeptides, also exhibit antifreeze properties (Figure 5) [48]. Therefore, studying proline and its derivatives is crucial for understanding low-temperature protection mechanisms. Polyproline (PPro), with its unique type II polyproline helix (PPII) [49] and amphipathic properties, can significantly slow down the growth of extracellular ice by forming a reversible bond with ice surfaces through its hydrophilic faces. This interaction prevents large ice crystals from directly damaging cells and mitigates the subsequent rise in osmotic pressure. For instance, in the protection of oocytes and human lung adenocarcinoma cells (A549), PPro can reduce the amount of DMSO needed and achieve excellent cell recovery rates (99% for oocytes, 53% for A549) [41,50]. However, it is important to note that the antifreeze activity of PPro is concentration-dependent, showing negligible antifreeze activity at lower concentrations [42]. The cryoprotective efficacy of PPro is not only dependent on concentration but also influenced by its molecular weight. Lower molecular weights (MW) correspond to lower IRI activity. This phenomenon was substantiated through a comparative examination of cryoprotective effects using PPro with different MWs (ranging from 1200 to 50,000 g/mol). PPro with a MW of 50,000 g/mol could inhibit the growth of almost all ice crystals at concentrations below 5 mg/mL, demonstrating stronger efficacy than 20 mg/mL PPro with a MW of 2000 g/mol [50,51]. In summary, as our understanding of PPro deepens, it emerges as a potential non-toxic alternative to DMSO in cryoprotection, especially with its superior performance linked to increased MW and concentration.

### 4.3. Antifreezing Peptides Based on Threonine

**Figure 6:**
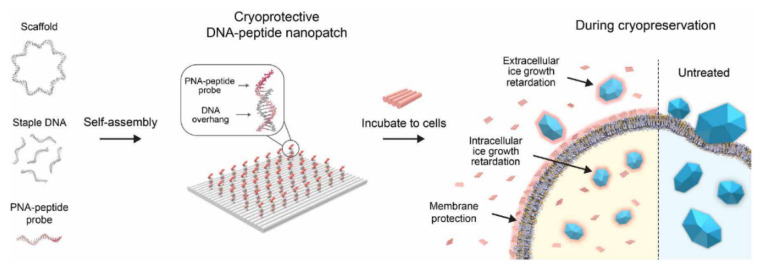
Schematic illustration of the assembly of cryoprotective DNA-peptide nano-patches and their preservation mechanisms.

Threonine (Thr), a hydrophilic amino acid, can form hydrogen bonds with ice, thereby conferring antifreeze properties. Thr is a common component in antifreeze peptide sequences, although it does not play a crucial role in AFPs. Researchers have investigated polythreonine (PThr), formed through self-assembly (Table 1). It was demonstrated that while the Ice Recrystallization Inhibition (IRI) activity of PThr (DP = 5) with spherical self-assembled structures is lower than that of PAla, it still achieves 40% of the Maximum Growth Limiting Size (MGLS) compared to PBS [44]. To further enhance the frost resistance of PThr, researchers constructed an orderly and spaced PThr that can adjust its side chains to optimize cryoprotective properties [52]. This modified PThr demonstrated cell viabilities of 82% for human liver cancer cells and 88% for normal liver cells [53]. Additionally, self-assembly with DNA nanosheets significantly improved the frost resistance of PThr by combining the expansive surface area of nanosheets with the frost resistance of PThr (Figure 6). Consequently, the single-layer Thr-DNA origami nano-patch enhanced the survival rate of human oral squamous cell carcinoma (HSC-3) to 56% after one month of freezing, surpassing the 38% survival rate achieved with the conventional cryoprotective agent DMSO [54].

### 4.4. Antifreezing Peptides Based on Lysine

Lysine, an amino acid known for its hydrophilic properties due to its innate positive charge, shows notable cryoprotective abilities [55]. A derivative of lysine called *ε*-poly-*L*-lysine (PPL), which features a carbonyl group at the α-position and an amino group at the ε-position, is a cationic polymerized polypeptide. Initially used as an antibacterial agent, PPL has recently gained attention for its cryoprotective properties [56,57]. Recent research has highlighted the potential of carboxylating the side chains of PPL to enhance its cryoprotective efficacy, making it increasingly useful in sperm cryopreservation. For instance, the rate of DNA breakage in human sperm can be kept below 30%, and fish sperm activity is boosted by 10% after thawing [58,59]. Moreover, the versatility of lysine’s unique side chain structure allows for various modifications that further improve its cryoprotective characteristics. Modifications involving poly(vinyl alcohol), succinic anhydride, and dodecenylsuccinic anhydride have demonstrated significant cryoprotective properties [60,61,62]. For example, when combined with poly(vinyl alcohol), PPL can maintain 78% of its lactate dehydrogenase activity, even after 15 cycles of repeated thawing. Additionally, when modified with dodecenylsuccinic anhydride, lysozyme retains up to 95% of its activity. These advancements underscore the potential of PPL and its modifications as effective cryoprotective agents, paving the way for broader applications in biopreservation.

### 4.5. Other Chemical Synthesis Antifreezing Peptides

**Figure 7:**
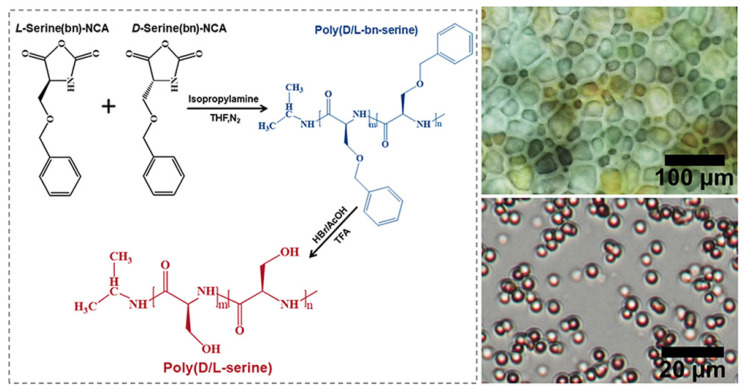
Schematic illustration and IRI of the Poly(*D*/*L*-serine) Synthesis.

In addition to the four main amino acids discussed previously, other polypeptide substances dominated by serine (Ser) also exhibit antifreeze properties. *D/L*-Polyserine, synthesized through NCA polymerization to copolymerize *D/L*-serine in various *L/D* ratios (7:3, 5:5, and 3:7) (Figure 7), has been found to be non-cytotoxic. Although its Ice Recrystallization Inhibition (IRI) activity levels (63.6%, 61.4%, and 59.7%, respectively) were not particularly significant, the hydrophilic nature and non-cytotoxic properties of Ser make it more effective than hydroxyethyl starch by 20%, and more effective than polyethylene glycol and polyvinylpyrrolidone by 10% in the cryoprotection of sheep red blood cells. The respective cell recovery rates were 52.3%, 50.6%, and 49.8% [63].

With the development of computer science, the computer-aided design of AFPs has become a reality [64,65]. Researchers studied a novel approach combining Statistical Package for the Social Sciences (SPSS) and polypeptide sequences derived from in silico predictions. This antifreeze peptide precursor sequence was sourced from the autolysate of white-hair rough shrimp (Trachypenaeus curvirostris) byproducts. Among a series of peptides, DEYEESGPGIVH (AFP-1) and EQICINFCNEK (AFP-2) emerged as promising candidates. The thermal hysteresis (TH) values of AFP-1 and AFP-2 at a concentration of 10 mg/mL were 1.37 °C and 1.57 °C, respectively [66]. Apart from computational methods, researchers have also drawn inspiration from the sequences of AFPs in psychrophilic microorganisms [67]. A novel antifreeze polypeptide, AVD (AVDAGTGNDELIIGGDVSG), was developed based on the ice-binding protein sequence of Marinomonas primoryensis. Substituting amino acid residues at the sixth (T→S), eighth (N→S), and eighteenth (S→X) positions of AVD revealed that the Thr6 and Asn8 residues were essential for its ice-binding capability, while the Ser18 residue synergistically enhanced the interaction with ice. At a concentration of 10 mg/mL, AVD exhibited notable cell recovery rates of 70.9% for mouse embryonic fibroblasts (NIH-3T3), 34.9% for mouse mononuclear macrophage leukemia cells (RAW264.7), and 95.7% for human poorly differentiated lung adenocarcinoma cells (GLC-82) [68].

## 5. Microbial Synthesis of Antifreezing Peptides

The generation of diverse bioactive peptides with varying amino acid sequences and molecular weights through microbial metabolism and fermentation under different conditions has a rich historical background. As a result, significant research has been dedicated to leveraging microorganisms for the production of antifreeze peptides. For instance, the prolonged low-temperature fermentation of yogurt by lactic acid bacteria has been associated with the production of antifreeze peptides [69,70]. However, after separation, these peptides often exhibit low purity and uncontrollable sequences, prompting researchers to employ genetic engineering techniques to introduce specific sequences into microorganisms for tailored antifreeze peptide synthesis. For example, the introduction of the gene encoding the snow flea antifreeze peptide (rsfAFP) into *Bacillus subtilis* [71] and *Escherichia coli* BL21(DE3) [72] has demonstrated the production of antifreeze polypeptides with significant low-temperature protective properties, notably against Streptococcus thermophilus, where cell viability reached 83% after thawing. Besides the rsfAFP gene, valuable sources such as *Tenebrio molitor* [73], *Pseudomonas borealis* [74], and marine organisms [75] have also been identified for encoding antifreeze proteins. Particularly noteworthy is the remarkable protective efficacy exhibited by antifreeze polypeptides derived from the Tenebrio molitor gene. The addition of just 0.5% of these peptides resulted in a substantial increase in the survival rate of NIH3T3 cells, from 37.86% to 81.32%.

**Figure 8:**
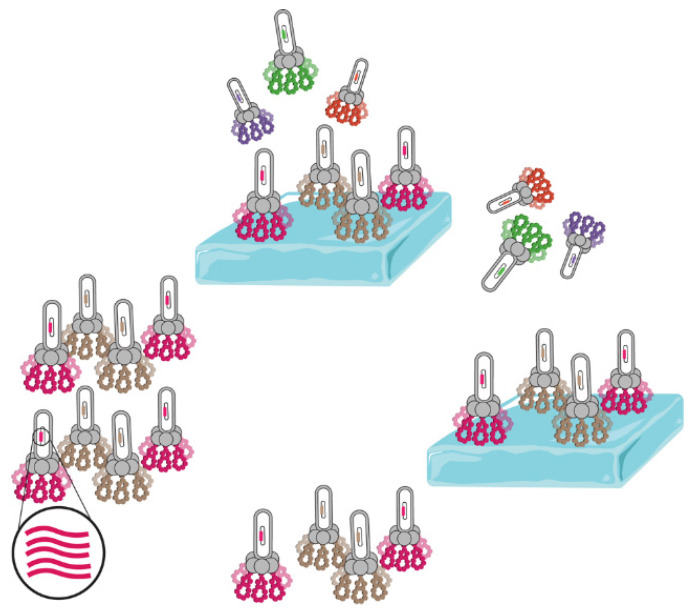
Schematic illustration of the phage display process used for the identification of ice-binding peptides.

Moreover, beyond bacterial synthesis, phages (Figure 8) have demonstrated the capability to synthesize cyclic antifreeze peptides (AFPs) with the main sequence ACSDRFRNCPADEALCG, thereby enhancing the feasibility of producing these compounds [76]. Consequently, microbial production emerges as a novel and valuable avenue for obtaining antifreeze peptides, showcasing the potential of genetic engineering and microbial systems in revolutionizing the production of biologically active molecules.

## 6. Other Antifreezing Materials

Despite the acknowledged limitations regarding the cytotoxicity associated with conventional cryoprotectants, the imperative to continue exploring these traditional solutions cannot be overstated. Advancing the field of cryoprotection requires a holistic perspective and dedicated efforts to overcome the drawbacks inherent in conventional protective agents. Consequently, ongoing research endeavors persist in evaluating the antifreeze capabilities of various compounds. Notable examples include polyvinyl alcohol, which achieves an 80–90% recovery rate for NIH-3T3 cells [77], polypropylene-based glycidyl ether [78], glycopeptide-like polymers that restore 87% vitality in frozen red blood cells [79], and polyampholytes, which show a recovery rate of 89% for A549 cells [80,81]. Each of these demonstrates promising low-temperature protective attributes for cellular systems.

## 7. Conclusions

In summary, this focused review highlights the current state-of-the-art in the rapidly emerging field of antifreeze peptides, which have the potential to surpass traditional antifreeze agents. Although research on antifreeze peptides is still in its exploratory stage, significant insights have been gained into how amino acids and secondary structures in their sequences influence antifreeze properties. This paper outlines various methods for obtaining antifreeze peptides: proteolysis, where AFPs are primarily derived from antifreeze proteins; chemical synthesis, using techniques such as SPSS, NCA, and self-assembly; and microbial synthesis, primarily employing genetic engineering. While proteolysis remains a common method, it faces several challenges, including random hydrolysis sites, unmodifiable peptide sequences, and a significant reliance on marine organisms. These issues often result in peptide mixtures unsuitable for specific applications and can exacerbate environmental pressures on ocean ecosystems. Microbial synthesis also encounters challenges related to gene design and introduction. Consequently, chemical synthesis emerges as the most promising solution for future AFP production due to its ability to accurately control the sequence of obtained peptides. Moreover, this review provides a detailed introduction to the amino acids, structural patterns, and primary sequences that play key roles in antifreeze peptides. It is intended to serve as a valuable reference for the future design and synthesis of antifreeze peptides.

## Figures and Tables

**Table 1:** Sequences and effects of valuable chemical synthesis antifreezing peptides.

Main Amino Acid	Sequence	IRI Activity (MGLS, Concentration)	TH Activity (°C, Concentration)	Cell Recovery Rate	Reference
Ala	AAAAA	17%, 1.0 mg/mL	1.05 °C, 1.0 mg/mL	N/A	[23]
	*L*-Ala-co-*L*-lys	11%, 10 mg/mL	0.2 °C, 1.0 mg/mL	N/A	[24,25]
	DTASDAAAAAAL	N/A	0.46 °C, 10 mg/mL	N/A	[26]
	KATATAKA	24.3%, 7.5 mM	N/A	N/A	[27]
Pro	Pro_n_ (*n* = 8)	30%, 36.84 mg/mL	N/A	99% of oocytes	[30]
	Pro_n_ (*n* = 15)	50%, 20 mg/mL	N/A	N/A	[31]
	Pro_n_ (*n* = 515)	9%, 5 mg/mL	N/A	N/A	[33]
Thr	TTTTT	40%, 1.0 mg/mL	1.09 °C, 1.0 mg/mL	N/A	[23]
	2-naphthylacetyl-G-F-F-PThr	N/A	N/A	82% of SMMC-7721, 88% of L-O2	[35]
	DNA-PThr	N/A	N/A	56% of HSC-3	[36]
Ser	*D*/*L*-Polyserine	59.7%, 60 mg/mL	N/A	49.8% of sheep’s red blood cell	[40]
Glu	DEYEESGPGIVH	N/A	1.37 °C, 10 mg/mL	N/A	[41]
Gly	AVDAGTGNDELIIGGDVSG	N/A	N/A	70.9% of NIH-3T3, 34.9% of RAW264.7, and 95.7% of GLC-82.	[42]
